# Cheating and plagiarism in higher education institutions (HEIs): A literature review

**DOI:** 10.12688/f1000research.147140.1

**Published:** 2024-07-10

**Authors:** Md Sozon, Omar Hamdan Mohammad Alkharabsheh, Pok Wei Fong, Sia Bee Chuan

**Affiliations:** 1 Universiti Tunku Abdul Rahman, Bandar Sungai Long, 43000 Kajang Petaling, Selangor, Malaysia

**Keywords:** students, cheating, plagiarism, higher education institutions, academic integrity.

## Abstract

Cheating and plagiarism have become serious problems in higher education institutions (HEIs). It affects educational quality as well as the reputation of higher education. The purpose of this study is to identify the most prevalent types of cheating and plagiarism, as well as the elements that contribute to cheating and plagiarism, and to present solutions to this recurring problem.

This paper systematically reviews 45 articles published from 2018, to 2022, aligned with the PRISMA guidelines in the selection, filtering, and reporting of the papers.

This review shows that factors such as increased pressure on students, poor academic integrity awareness, lack of up-to-date academic honor codes, and the unethical application of AI tools are prime contributing factors to cheating and plagiarism in HEIs. In a broader sense, all these factors are classified as individual, social, cultural, institutional, and technological factors that are responsible for this problem.

This problem can be reduced by establishing ethical and moral development tutorials as well as formulating up-to-date honor codes considering AI tools. Furthermore, higher education institutions must develop anti-plagiarism detection software in order to detect plagiarism and aid students in improving academic writing and paraphrasing approaches.

The findings of this systematic literature review provide useful insights for educators and policymakers to solve the complicated issue of cheating and plagiarism in higher education institutions.

**List of Abbreviations**
HEIs: Higher education institutions
ICAT: International Center for Academic Integrity
ChatGPT: Chat Generative Pre-trained Transformer
AI: Artificial intelligence
SLR: Systematic Literature Review
PRISMA: Preferred Reporting Items for Systematic Reviews and Meta-Analyses
IC: Inclusion criteria
EC: Exclusion criteria
N: Number
RoB2: Risk of bias
D1: Bias arising from the randomization process
D2: Bias due to deviations from intended intervention
D3: Bias due to missing outcome data
D4: Bias in measurements of the outcome
D5: Bias in the selection of the reported

## 1. Introduction

Academic integrity is an essential element for higher education institutions (HEIs) all over the world ( Vasylkevych & Lomak, 2020; Hölscher, 2021). Academic integrity is a commitment not only to uphold ethical values, but also to demonstrate behaviors that promote six fundamental principles *“honesty, trust, fairness, respect, responsibility, and courage*” in all aspects of education, research, and scholarly communication within the context of higher education ( International Center for Academic Integrity [ICAI], (2021). In this sense, 97% of guardians in the United States of America believe that moral values such as honesty and integrity should be incorporated into the academic curriculum ( Pavela, 1993; Shrivastava, 2017) and higher education institutions are responsible for promoting academic integrity ( Mattar, 2022) and producing ethically and morally sound graduates ( Alhamuddin et al., 2023).

However, higher education institutions around the world are struggling to maintain academic integrity due to the recurrent occurrence of cheating and plagiarism. Cheating and plagiarism are prevalent problems in HEIs that threaten the core principles of education and undermine the student's ethical and moral values ( Sozon et al., 2024). The availability of technology-driven artificial intelligence tool, for example, Chat Generative Pre-trained Transformer (ChatGPT) supports to violate academic integrity ( Susnjak, 2022) and corrupts user judgment ( Krügel et al., 2023), raises concern about ethics and professionalism ( Currie, 2023), promotes high-tech plagiarism ( Gefen & Arinze, 2023), students not considered as high-risk technology tool about cheat ( Fergus et al., 2023), teachers find hard to detect the authenticity of students assignments and exams ( Crawford et al., 2023), users may publish the paper as their own ( Oravec, 2023), raises ethical and privacy issues ( Shahriar & Hayawi, 2023) and sparks controversy among higher education community. In the future, AI is more likely to develop ( Tomlinson et al., 2023) and produce more customized educational materials, which may disrupt students' motivation for acquiring knowledge and higher education institutions' learning environment. Moreover, it may also discourage students from developing new skills and abilities by putting in an effort and investing time to sharpen their expertise. AI tool (ChatGPT) is a potential threat to academic integrity in all levels of higher education ( Williams & Fadda, 2023) because all types of cheating raise questions regarding the quality of an institution's academic curriculum, the value of its degrees, and the skill of its graduates ( Wideman, 2008).

This scope of this study covers various forms of academic integrity violations, from classic exam cheating to sophisticated cases of plagiarism made possible by digital technology. It is crucial to recognize that combating academic misconduct such as cheating, and plagiarism requires a variety of approaches. The authors intend to examine the strategies and interventions that universities can implement to combat cheating and plagiarism. Therefore, understanding the effectiveness of these measures is critical for institutions seeking to protect the integrity of their academic programs.

With this systematic literature review, the authors aim not only to comprehensively synthesize existing knowledge but also to identify gaps in the literature, thereby paving the way for future research efforts. The authors also consider the scope of different studies, the geographical location of the selected papers, research methods, their impact on citations, reasons for cheating and plagiarism, and ways to prevent cheating and plagiarism in higher education institutions and contribute to the ongoing discourse on academic integrity, providing educators, administrators, and researchers with valuable insights to inform evidence-based policies and practices.

The four research questions listed below served as the study’s compass in meeting its objectives and identifying the untapped research fields:

1. What are the characteristics of research and publication on the prevalence of plagiarism and cheating in HEIs (volume of publications, pattern of papers, field of study, etc.) of the reviewed papers published from 2018 to 2022?
2. What are the top prevalent types of plagiarism and cheating in HEIs?
3. What factors contribute to the prevalence of cheating and plagiarism in Higher education institutions?
4. What strategies have been employed to reduce the prevalence of cheating and plagiarism in HEIs

This research will provide evidence-based guidance to educators and other responsible stakeholders for the formulation and modification of academic integrity policies and procedures within educational institutions to combat students’ inappropriate academic behavior (cheating and plagiarism). This study will also explore the design and development of plagiarism detection software and innovation assessment systems that encourage critical thinking, originality, and ethical research practices. Thus, the purpose of this literature review is to explore the existing body of knowledge and summarize the results of various academic studies to reveal a comprehensive picture of the prevalence of cheating and plagiarism in higher education institutions.

## 2. Literature review

Cheating and plagiarism are prevalent issues in higher education institutions around the world. HEIs have become increasingly concerned about the prevalence of cheating and plagiarism in recent years. It has further increased due to the unethical application of artificial intelligence tools (AI) in manipulating the examination and writing assignments, reports, case studies, thesis, and research papers. The scholarly publication shows that students are easily able to cheat and plagiarize written assignments through inappropriate applications of technology and AI tools.

Cheating is defined as the use of data, resources, equipment, or methods for the completion of academic tasks in a dishonest manner ( Salamah, 2022). The duplication and submission of the work of another student without their permission also come under the umbrella of cheating and it describes the exploitation of prohibited materials, work plagiarizing, or dishonest behavior such as telling lies of being ill or injured as leverage in tests ( Hodgkinson et al., 2016). In short, cheating is often adopted to gain an unfair advantage over other students from the teachers.

Cheating is a growing concern in higher education institutions. In the past, many scholarly papers were published to identify and provide solutions to this problem. However, this problem bv hgbvbv cheating.

On the other hand, the term plagiarism stems from the root Latin term “plagiarism” which means “kidnapper/abductor”. ***Plagiarism** is the* unauthorized use of a source in situations where there is a legitimate expectation of authorship. Plagiarism is using another individual’s thoughts, words, phrases, sentences, or facts and taking them as one’s own ( Thomas, 2020). According to Sutton et al. (2014) plagiarism is considered as academic dishonesty or contribution to misleading while taking credit for work that is owned by someone else. In the same way, other authors ( Farook et al., 2020; Mbutho & Hutchings, 2021) defined it as a type of academic dishonesty that is considered fraudulent behavior, undermining the intellectual property of the author and obtaining a reward for another’s work. Viewed from a legal perspective, plagiarism is an act of theft of intellectual work ownership ( Gullifer & Tyson, 2010, p. 463). It is a violation of intellectual property rights that are protected through copyright laws. It appears that plagiarism has legal as well as ethical ramifications and is sometimes viewed as a violation of moral-ethical aspects as opposed to legal aspects owing to its nature of being outside of the copyright infringement rights boundaries ( Mbutho & Hutchings, 2021).

Plagiarism is the top frequently used method in academia which breaches the integrity of HEIs. According to the opinion of ( Butakov et al., 2012), this has become a huge concern in several fields, namely education, research, and industry and it is deemed to be a significant misbehavior breaching academic ethics and intellectual thought. Moreover, ( Gullifer & Tyson, 2010) described the issue as increasingly worsening among institutions, urging them to focus more on its resolution. The issue is being focused on due to the increasing prevalence of plagiarism in the technological era, aiding students in claiming someone else’s work as their own. Information and communication technologies (ICT) development has led to the promotion of plagiarism among students at higher educational institutions ( Rozar et al., 2020). Stealing another’s work breaches the fundamental foundations of the academic community ( Helgesson & Eriksson, 2015; Eret & Gokmenoglu, 2010) as it is dishonesty, and a normal and decent person possessing morals and values would never engage in plagiarism behavior.

Various sources are attributable to the rise of plagiarism, among which are the failure to cite sources properly, honest mistakes, and divergent views on what comprises suitable academic behavior/scholastic integrity in different cultures ( Sarlauskiene & Stabingis, 2014). This stresses the consideration behind the phenomenon’s motivation when addressing the issue and the fact that evidence-based reasoning may not be effective in clarifying the low levels of students who are penalized or expelled from educational institutions for plagiarizing. This era of internet technology has abetted fraudulent and corrupt behaviors among higher learning institution students, which has become a source of concern. Consequently, the increasing and extensive prevalence of plagiarism in several colleges of industrialized nations has focused on technology to prevent the behavior among students ( Thompsett & Ahluwalia, 2010). Regardless of such technological detection of plagiarism, institutions in developed nations are still not leveraging effective plagiarism detection software. Therefore, further study is essential to determine the contributing factors of student plagiarism and design a solution to solve this burning issue.

Cheating and plagiarism in universities have far-reaching consequences. It not only disrupts classroom decorum but also affects students' ethics and professionalism. Cheating and plagiarism can hurt educational institutions, teachers, and learners both domestically and internationally ( Pettyjohn et al., 2020). Students who commit academic dishonesty in their educational institutions are likely to continue to engage in similar unethical activities in their professional lives ( Ozcan et al., 2022). Moreover, violations of students' academic integrity harm not only students' learning outcomes but also the reputation of educational institutions. Furthermore, students' cheating and plagiarism may lead teachers, prospective students, and researchers to engage in similar inappropriate activities in their fields ( Waigand, 2019). Therefore, every effort should be made to detect and prevent students' cheating and plagiarism that affect various stakeholders.

Higher education institutions must address cheating and plagiarism issues seriously. Institutions must put in place effective rules and processes to ensure academic standards and integrity Darman, 2022). To address these challenges, institutions must deploy robust plagiarism detection tools, secure online exam platforms, and clear academic integrity policies, while also fostering a culture of ethical AI use and digital literacy among students ( Jenkins et al., 2022). Striking a balance between the benefits and risks of AI in higher education requires a comprehensive and proactive approach to maintaining academic integrity and upholding the ethical standards of education. Failure to do so will destroy the inclusive learning environment designed for ethical practice. Higher education institutions are also required to initiate a combination of educational, technical, and supportive measures to reduce recurrent incidents ( Abbasi et al., 2021). One potential solution to address this issue is to increase awareness about the negative consequences of cheating and plagiarism ( Kier & Ives, 2022). Another solution is to implement stricter consequences for those who engage in these practices. Additionally, encouraging ethical behavior through education and mentorship can help prevent cheating and plagiarism ( Mutongoza & Olawale, 2022). The solution aims to promote a culture of integrity, responsibility, and intellectual growth, and ensure the integrity of the educational experience for all involved. All stakeholders must work closely together to foster a culture of academic integrity in higher education institutions.

Overall, the literature review highlights the significance of sustaining educational ethics and taking action to prevent and address cheating and plagiarism in HEIs. Institutions and individuals must prioritize academic integrity to ensure that students receive a quality education and obtain legitimate qualifications. This research may help to address the challenges relating to students cheating and plagiarism behaviors in educational contexts by offering useful findings to education institutions, policymakers, and professionals. Furthermore, this study will reinforce the necessity to upgrade the existing plagiarism detection software and make it more diversified to detect AI and software-produced write-ups. In summary, this study is critical given the present state of higher education, and it will significantly advance our knowledge of students' unethical behavior in the classroom and teach them to uphold the institution's code of ethics.

## 3. Methods

A systematic literature review (SLR) refers to a process involving analyses, condensing, and interpretation of research inferences according to sources, while complying with the established rules ( Tawfik et al., 2019). This study follows PRISMA framework (Mckenzie, et al., 2021; Moher, et al., 2021; Sozon et al., 2024). The PRISMA framework presents a core foundation for the systematic articulation and visualization of the way the study results are acknowledged and reached during the process of review and meta-analysis ( Surahman & Wang, 2022). SLR is justifying the conditions for information sources utilized, the procedures of collecting data, providing descriptions of data and reaching conclusions.

### 3.1 Data source and search techniques

This study considered articles published from2018, to 2022, in the Scopus database. This database was chosen due to its availability and easy access to internationally recognized articles including peer-reviewed papers, and scientific literature published worldwide and in various disciplines. The search technique centred around key concepts linked to the subject of study topic (cheating, plagiarism, academic dishonesty, academic misconduct, academic integrity violation in Higher education institutions” OR “tertiary education institutions), and simple operators’ Boolean operators (AND, OR) were used based on a research question.

The study was limited to peer-reviewed articles, which helped it become more reliable and comprehensive. As the search continues, additional parameters are used in the database to search based on the inclusion and exclusion criteria described in section 3.2, To provide the broadest possible overview, no specific journals were selected during the search strategy stage. In the continuation of the search, additional parameters (document type: articles, review articles; language: English) to refine the search results were used in the database based on the inclusion and exclusion described in section 3.2. To offer the widest possible view, particular journals were not chosen during the search strategy phase.

### 3.2 Inclusion and exclusion criteria

Clear inclusion and exclusion criteria were established to choose and incorporate only pertinent papers that were found in the database related to our study subject.

3.2.1 Inclusion criteria

IC1: Journal articles published in Scopus Database only.

IC2: Research papers published between 2018, and 2022.

IC3: The research paper is written in English Language only.

IC4: The research is related to the Social Sciences, Arts and Humanities, and Psychology field.

IC5: The study was carried out in an educational setting (higher education institutions, tertiary education).

IC6: Only available complete papers.

IC7: The research address (cheating, plagiarism, academic dishonesty, academic misconduct, academic integrity violation).

**3.2.2 Exclusion criteria**

EX1: Journal editorials, books, book chapters, conference article and other nonpeer-reviewed publications.

EX2: The journal paper is not written in English Language.

EX3: The journal paper is not peer-reviewed

EX4: The journal paper is listed in other databases.

EX5: The research is not conducted in an education environment (higher education institutions, Tertiary education).

EX6: The journal paper is not related to the Social Sciences, Arts and Humanities, and Psychology field.

EX7: The study does not address (cheating, plagiarism, academic dishonesty, academic misconduct, academic integrity violation).

EX8: Non-availability of complete peer-reviwed papers

EX9: The articles published beyond the selected time frame between 2018, and 2022.

EX10: Duplication of data: The search strings generated the same papers more than once.

### 3.3 Data collection and analysis

**Figure 1.**
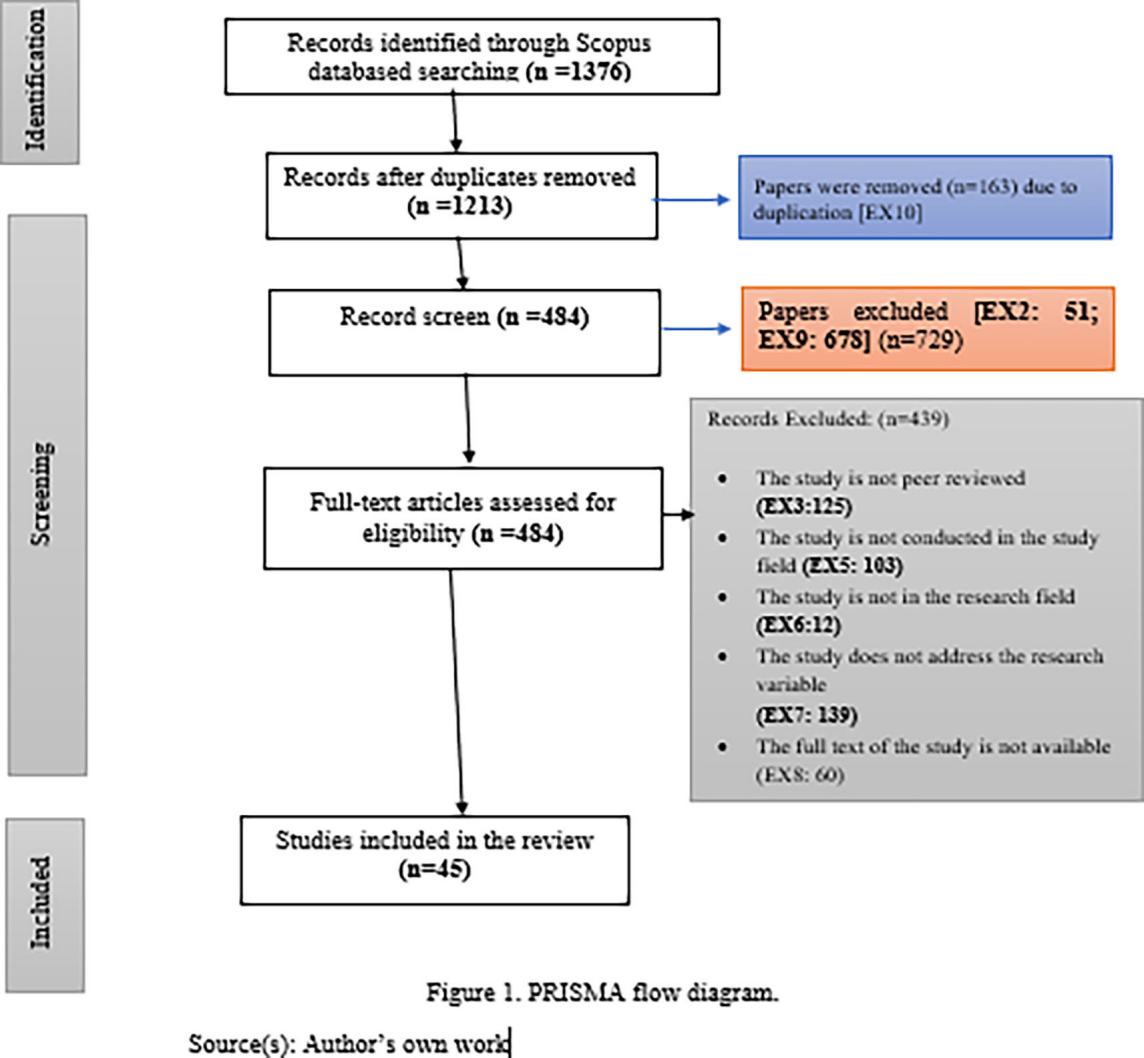
PRISMA flow diagram.

The systematic literature review was carried out in five phases by two researchers following the PRISMA guidelines. The first phase consisted of an initial search of the literature included in the Scopus Database (n=1376). In the second phase, a total of 163 studies were dropped because of duplication of data **(EX10).** A total of 729 papers were eliminated in the third phase based on the exclusion criteria **(EX2: 51; EX9: 678).** Subsequent stage, A total of 439 papers were dropped due to the above-written exclusion criteria **(EX3:125; EX5: 103; EX6:12; EX7:139; EX8:60).** In the fifth phase, consequently, 45 articles were selected based on the inclusion criteria. A list of selected papers is shown in PRISMA flow diagram ( Figure 1). Moreover, all the selected 45 articles were publications in 34 well-known global index journals for the years 2018-2022, distributed as follows 7 in 2018 (16%), 2 in 2019 (4%), 7 in 2020 (16%), 10 in 2021 (22%), and finally, 19 in 2022 (42%). Besides, these papers also encompass an extensive array of academic subjects, from Social Sciences (84%) to Psychology (7%) and Humanities (9%).

The 45 papers that were included in the SLR are available in Extended data file 3. All 45 eligible articles were entered into Microsoft Excel and alphabetically arranged based on authors’ names. Each article was then coded serving as a building block for the content analysis method.

### 3.4 Synthesis and data analysis

Research findings were thoroughly examined by going through the discussion section, after which thematic and sub-thematic data analysis approach was utilized. Thematic analysis is an analysis that determines new themes and sub-themes. This ensured a better and more organized presentation of results. After a manual analysis method through content analysis strategy, the direction of future studies was mapped out by reading the discussion and conclusion sections of the articles. In a qualitative data analysis, such as this study, the acquisition, compilation, and evaluation of data is conducted in a non-numerical and unstructured method using a grounded theory method. The articles were categorized based on the study question topic, namely, to reduce cheating and plagiarism in HEIs. Data was arranged based on the questions following the manual analysis method which constitutes qualitative data acquisition, data organization and connection to the research subject, data coding and analyzing insights, and drawing up the findings in the form of a report.

### 3.5 Risk bias assessment

**Figure 2.**
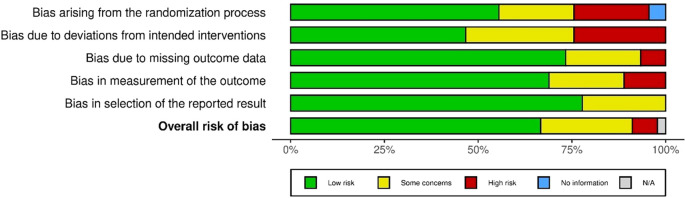
Summary of risk bias assessment (n=45) ( *McGuinness & Higgins, 2020*).

To ensure the authenticity, reliability, and credibility of the research and included paper for this study, the authors conducted a risk bias assessment (see Figure 2) by applying the Cochrane ‘ *Risk of bias’* tool for randomized trials (RoB 2.0) ( McGuinness & Higgins, 2020). A bias is a “systematic error, or deviation from the truth, in results or interferences”. A study may be at risk of bias due to issues with the conceptualization, design, conduct, or interpretation of the study and this may lead to misleading estimates of effect

In this process, the authors tried to address the five specific domains where the biases may occur: “ *bias arising from the randomization process, bias due to deviation from intended intervention, bias due to missing outcome data, bias in the measurement of the outcome, and bias in the selection of the reported results*”. Two authors (i and iv) independently applied this tool to each included study to provide supporting information and rationale for assessing the risk of bias in each area (low, high, and moderately concerning). Any conflicts in the decision that risked biasing or justifying the verdicts were resolved through discussion to reach an agreement The authors created a risk of bias summary (low, moderately concerned, high) for each included article Finally, the authors used the Robvis software to summarize the risk assessment and create a risk chart for this systematic review, as shown in Extended data file 2.

This review is therefore fully transparent and able to provide free, fair, natural, and reliable research results to resolve issues related to violations of academic integrity in higher education institutions.

### 3.6 Reporting the review

The findings in the articles concerning cheating and plagiarism among HEIs were examined in a systematic literature review in compliance with the PRISMA paradigm, specifically ensuring that the inclusion and exclusion criteria were met Extended data file 1, along with data searching and selection, and the synthesis of the results. Based on the research questions, the result section provided a summarized version of each answer. The results have implications for educators and researchers as to the creation and implementation of effective practical solutions for minimizing the incidents of cheating and plagiarism among HEIs.

## 4. Results

**Figure 3.**
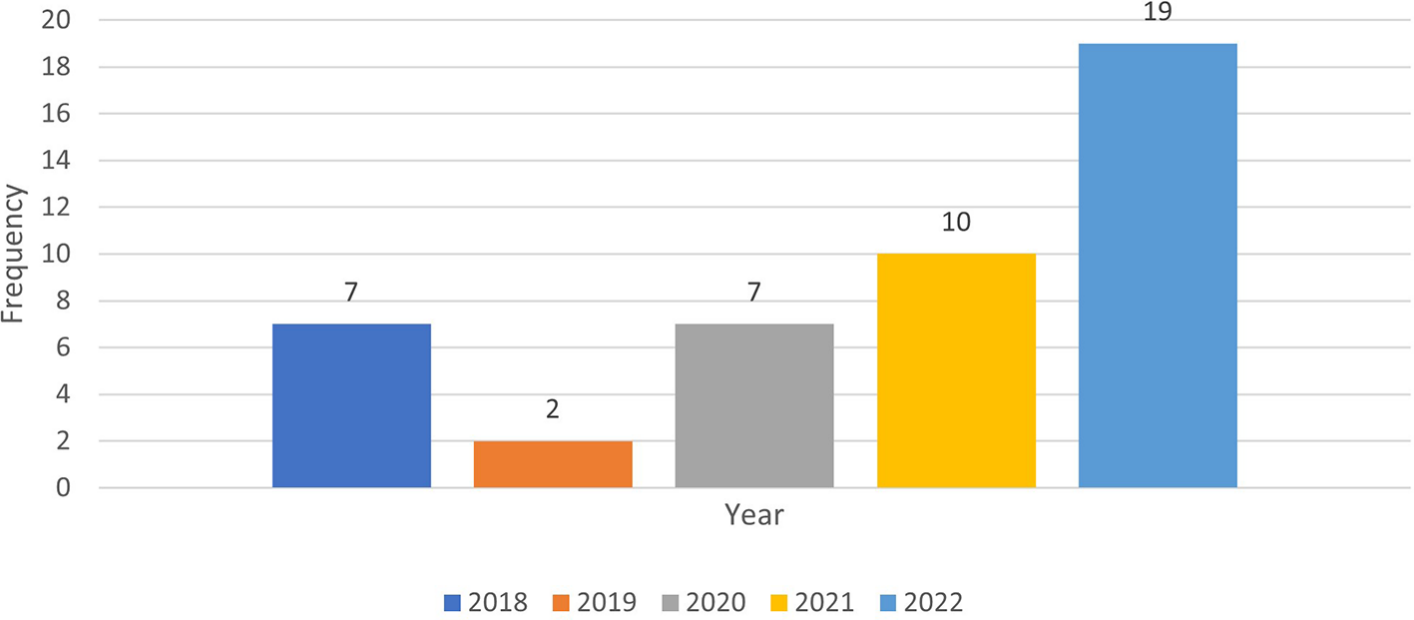
Frequency of publication each year (n=45).

The column graph (see Figure 3) shows the annual published papers selected for this review from 2018 to 2022.

The data represent the frequency of publication of cheating and plagiarism for the years 2018 through 2022. To compare the periods before and after the onset of the COVID-19 pandemic (which began in late 2019), we can split the data into two groups: pre-COVID (2018-2019) and post-COVID (2020-2022). It seems that the frequency of publications on cheating and plagiarism increased (80%) in the post-COVID period compared to the pre-COVID period (20%). This could be due to various factors, such as changes in educational settings, increased reliance on online learning, or other pandemic-related challenges.

**Figure 4.**
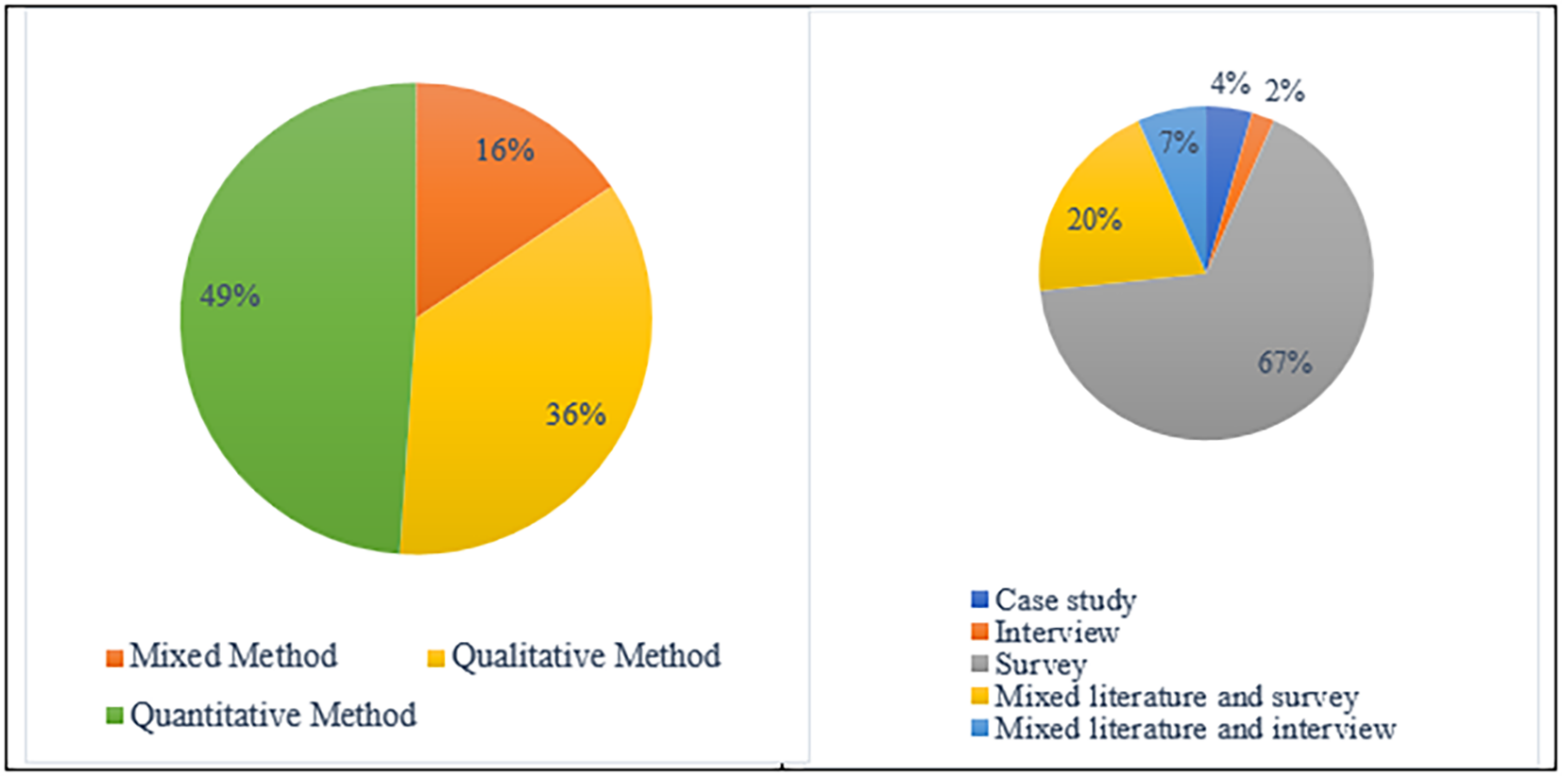
Frequency of publication research methods.

**Figure 5.**
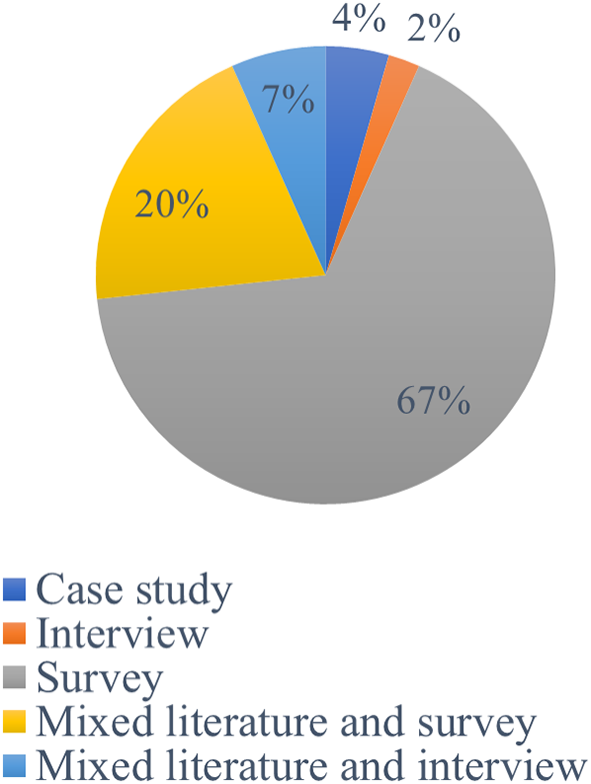
Data collection techniques (n=45).

In terms of methodological point of view **,** a variety of research approaches ( Figure 4) were used by earlier scholars to study academic integrity violations. According to the findings, 49% of the studies were based on qualitative research methods, while 36% of studies used quantitative research methods. Furthermore, mixed research approaches were used in 16% of the investigations. Figure 5 displays the methods mostly utilized to obtain research data: Casy study (4%). Survey (67%), Interview (2%), Mixed Literature and Interview (7%), and Mixed Literature and Survey (20%).

**Figure 6.**
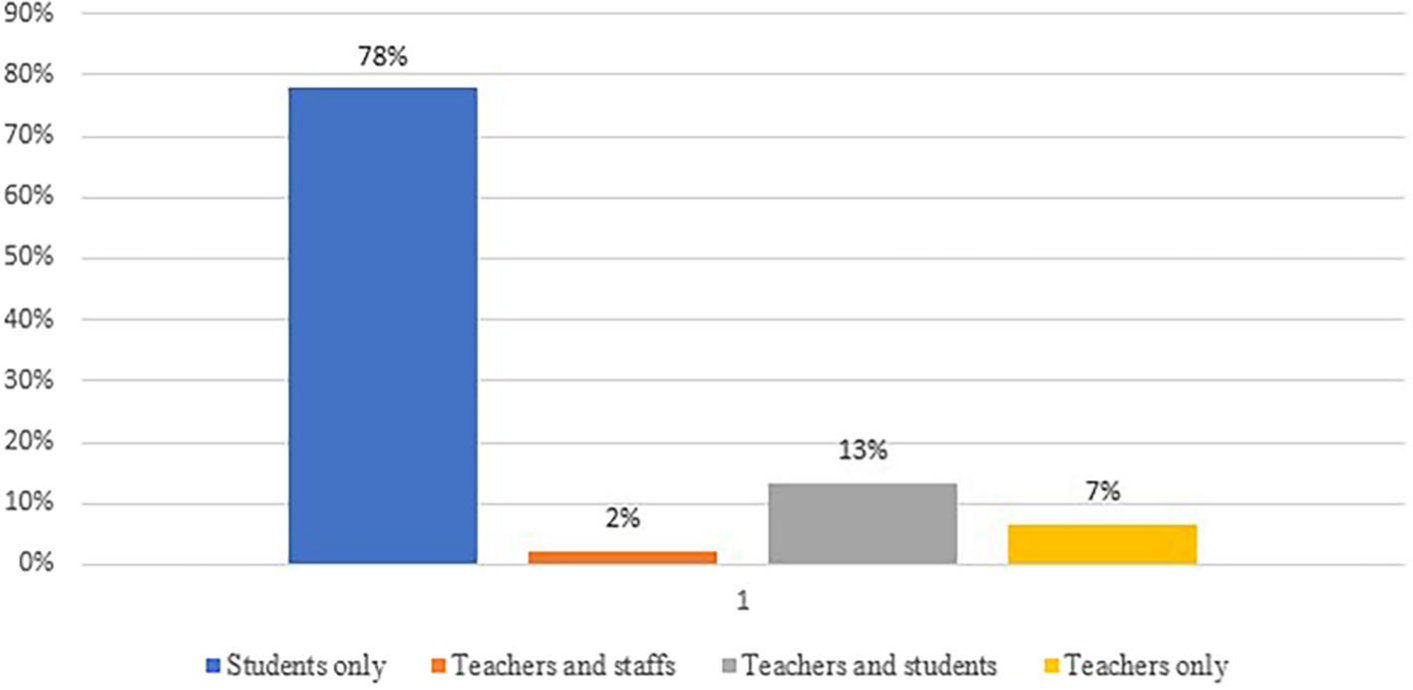
Frequency of publication each year focusing respondents (n=45).

According to the study, researchers have previously investigated a variety of topics to identify the root causes of academic integrity violations. To pinpoint the cause of this problem, they conducted tests on students, teachers, and staff (see Figure 6). The majority of research is centered around students (78%), indicating a significant emphasis on understanding their experiences, motivations, and methods related to cheating and plagiarism. Teachers and staff collectively constitute a small percentage (2%) of the respondents. This suggests that, compared to students, there may be less emphasis on exploring the perspectives of educators and administrative staff in cheating and plagiarism research. The category of both teachers and students (13%) reflects an interest in investigating the relationships and interactions between these two groups. This may involve studying how educators perceive and address cheating among their students. The subset of teachers-only (7%) respondents indicate a targeted focus on understanding the role of educators in the context of cheating and plagiarism, potentially exploring their awareness, preventive measures, and reactions.

***Trends in authorship analysis***

The analysis focused on authorship patterns that represent breaches of academic integrity in higher education research. The authors read and evaluated the articles. As shown in Table 1, analysis showed that 7% of the database's articles were produced by female scholars, whereas 51% of the articles were published by males. 42% of the papers featured both male and female scholars. Additionally, the study revealed that 29% of the publications had a single author and 71% of the papers had two or more authors.

**Table 1.** Analysis of trends in authorship (n=45).

Authorship		Gender authorship		
Single-authored	Co-authored	Male	Female	Mixed
29%	71%	51%	7%	42%

***The distribution of articles by selected journals***

**Figure 7.**
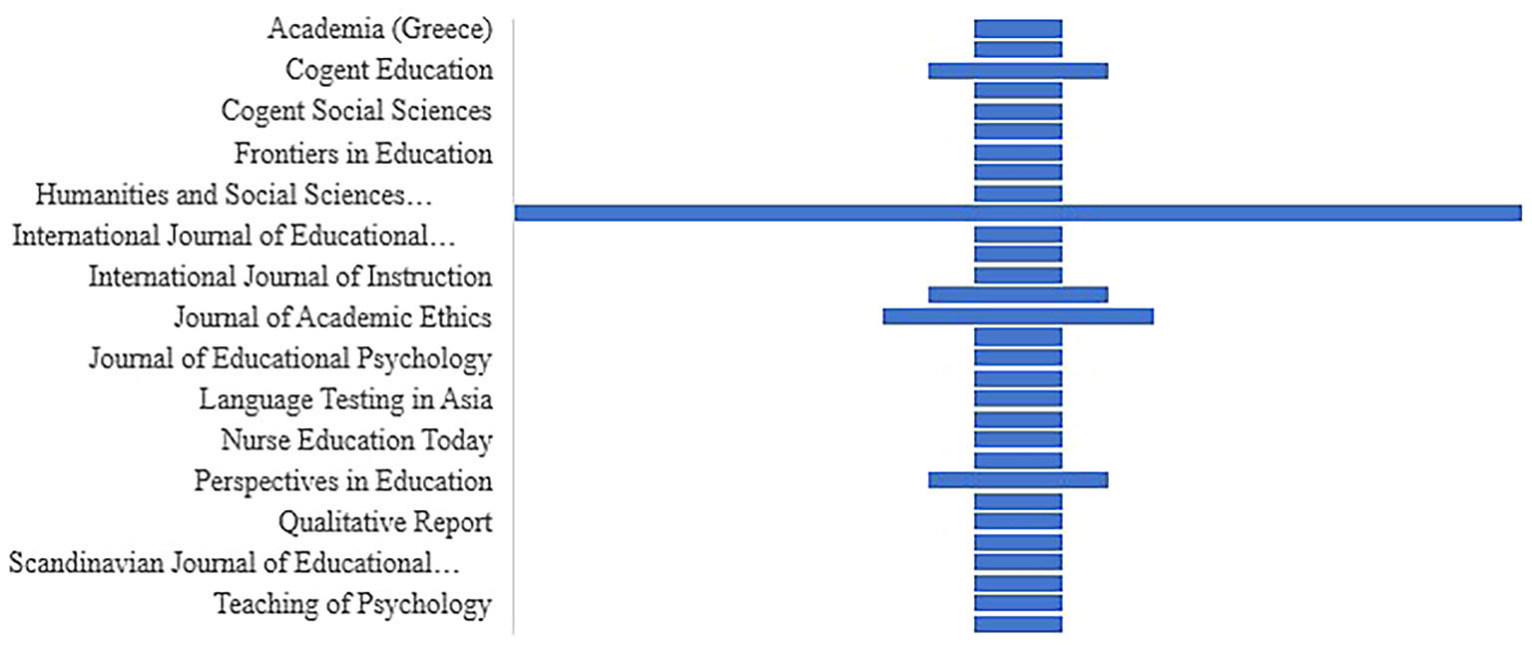
The distribution of articles by selected journals (n=45).

Between 2018 and 2022, a sizable number of research papers were published in high-impact journals (see Figure 7). The researcher had expressed great interest in publishing articles on cheating and plagiarism.

***Analysis of citation impact of the selected papers***

**Figure 8.**
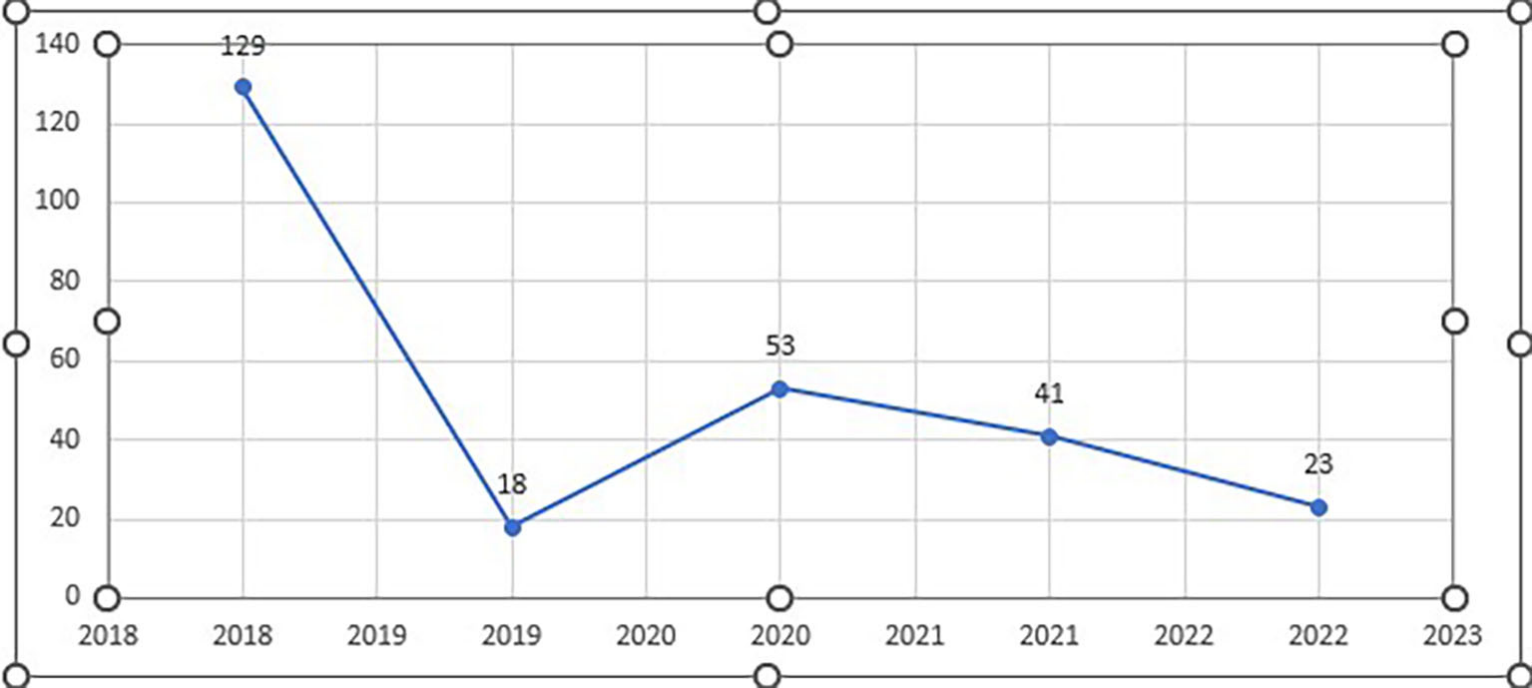
Year-wise number of citations (n=45).

The citation count exhibits some fluctuation from 2018 and 2022, suggesting that interest or effect levels were not constant over this time. Nonetheless, in comparison to the years that surround them, 2018, 2019, and 2021 stand out with comparatively larger citation counts. Perhaps throughout these years, there has been a rise in interest in the work or its increasing significance in the area (See Figure 8).

The highest citation count was observed in 2018, suggesting a peak in the impact or interest in research during that year. There was a substantial drop in citations in 2019 followed by a gradual increase in 2020, and a subsequent slight decrease in 2021 and 2022. The fluctuating pattern in citation counts across the years could be influenced by various factors, including the novelty and significance of the research, changes in the academic landscape, or the emergence of competing works.

***Publication of papers selected papers across countries***

**Figure 9.**
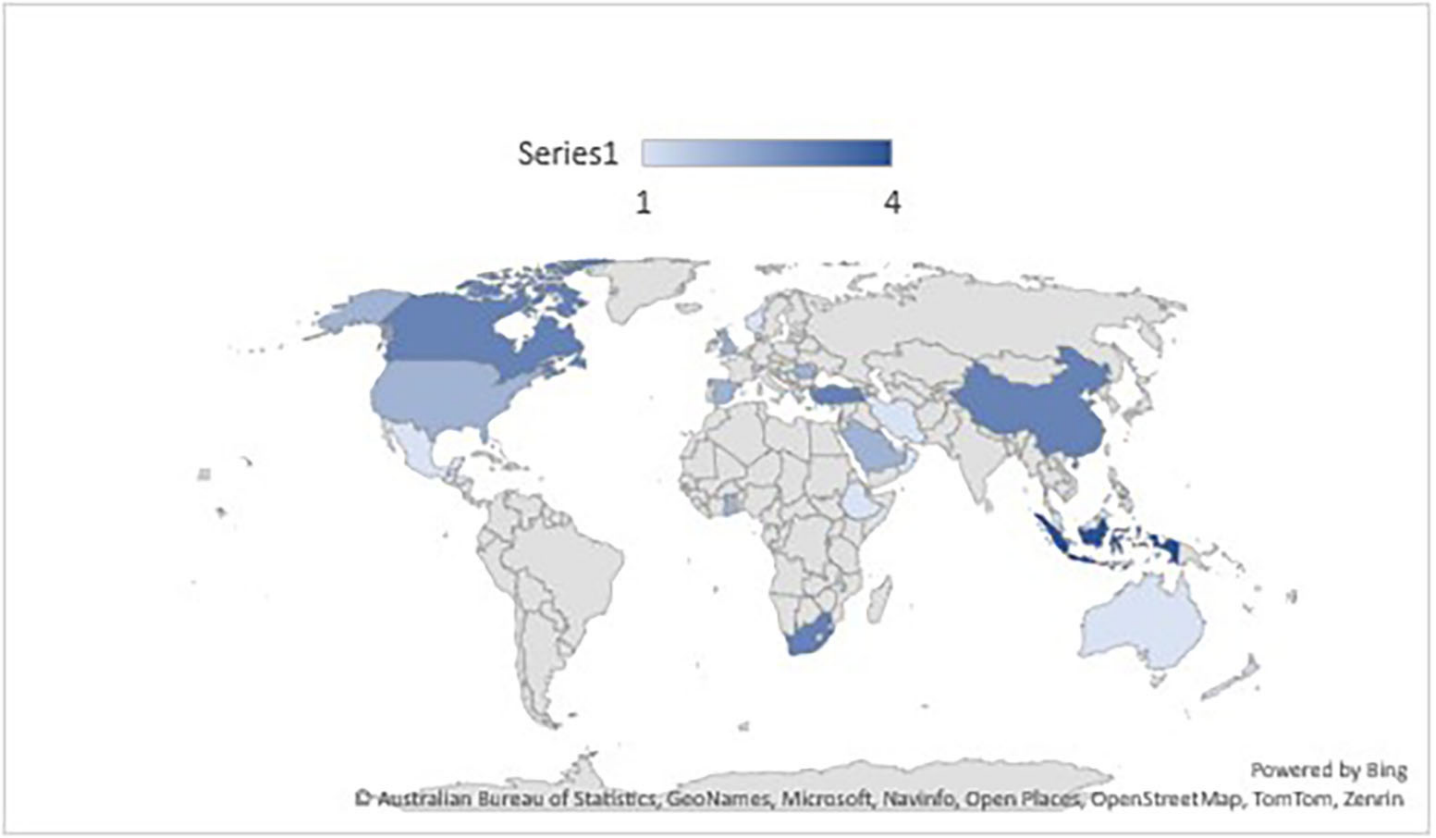
Frequency of publication according to the countries (n=45).

A map was produced to determine the source of publications in every nation, and it was categorized by the specific location of data collection. The map shows 45 papers were selected which originated from 28 different countries. More publications were from such as Turkey (n=3), USA (n=2), Indonesia (n=2) Canada (n=2), China (n=2), Saudi Arabia (n=2), South Africa (n=2). Other publications (n =30) were distributed across 21 countries *i.e., “Kosovo, New Zealand, Australia, Ethiopia, Ghana, Greece, Honduras, Iran, Israel, Kuwait, Malaysia, Mexico, Oman, Qatar, Romania, United Kingdom, Norway, Rwanda, and Switzerland, United Arab Emirates”* with one paper for each country (See Figure 9).

From the available statistics, it is clear that a varied range of nations appear to be represented, with each producing a distinct quantity of articles related to academic integrity violations. These review articles may certainly contribute to reaching an actionable solution for achieving this research objective.

**What are the top prevalent types of plagiarism and cheating in HEIs?**

In higher education institutions, academic dishonesty has grown in importance recently, especially when it comes to cheating and plagiarism. Research has indicated a rise in the frequency of cheating and plagiarism, particularly since the use of electronic assessments. This unethical behavior erodes the principles of academic honesty and jeopardizes the integrity of the educational system. The review of 45 articles following cheating and plagiarism forms in HEIs is tabulated in Table 2.

**Table 2.** Forms of Cheating and Plagiarism (n=45).

Form of cheating and plagiarism in HEIs	Number of review studies (percentage)	Study numbers based on the order of the table
Cheating	75.55%	E1, E3, E4, E6, E7, E9, E10, E11, E12, E13, E14, E15, E16, E17, E18, E19, E20, E22, E23, E24, E25, E26, E29, E32, E33, E35, E37, E39, E40, E41, E42, E43, E44, E45 (34)
Contract cheating	11.11%	E1, E5, E11, E21, E36 (5)
Collusion	13.33%	E5, E6, E11, E14, E21, E24 (6)
Copying	6.67%	E14, E19, E24 (3)
Academic misconduct	2.22%	E9 (1)
Falsification	2.22%	E7(1)
Fabrication	2.22%	E23(1)
Fraudulence	2.22%	E28(1)
Corruption	2.22%	E29(1)
Absenteeism	2.22%	E22 (1)
Plagiarism	42.22%	E2, E7, E8, E10, E11, E13, E14, E19, E21, E23, E27, E28, E29, E30, E31, E34, E35, E38, E43 (19)

**What factors contribute to the prevalence of academic cheating and plagiarism in higher education institutions?**

In higher education institutions, cheating and plagiarism have become prevalent issues. It's concerning because these immoral actions compromise the integrity of education and diminish the hard work of sincere learners. Academic cheating and plagiarism are common in higher education institutions for several reasons. Table 3 summarizes the key factors contributing to cheating and plagiarism in higher education institutions.

**Table 3.** Factors lead to cheating and plagiarism (n=45).

Factors lead to cheating and plagiarism	Reference Articles
Individual Factors	E2, E3, E4, E5, E9, E10, E12, E17, E18, E24, E26, E35
Institutional Factors	E1. E2, E4, E10, E12, E18, E25, E33
Social Factors	E2, E27, E31, E32, E33, E41, E44
Cultural Factors	E12, E29, E33
Technological Factors	E2, E35

**What strategies have been employed to mitigate academic cheating and plagiarism in higher educational institutions?**

Reducing the prevalence of cheating and plagiarism in Higher Education Institutions (HEIs) involves implementing a combination of preventive measures, educational initiatives, and disciplinary actions. Here are some common strategies that have been employe. The article review concerning plagiarism and cheating in HEIs also indicated the way they can be mitigated (see Table 4).

**Table 4.** Strategies to solve cheating and plagiarism (n=45).

Strategies to solve cheating and plagiarism	Reference Articles
Modification of academic integrity policy and honor code	E2, E3, E4, E5, E9, E10, E12, E17, E18, E24, E26, E35
Academic Integrity Awareness Program	E1. E2, E4, E10, E12, E18, E25, E33
Anti-plagiarism detection software	E2, E27, E31, E32, E33, E41, E44
Counselling and guidelines	E12, E29, E33

## 5. Discussion

This systematic literature review was conducted to provide an overview and insight into the research literature on cheating and plagiarism in HEIs to improve the quality of education and help students to develop ethical and moral skills. In this study a total of 45 papers ***(see Extended data file 3)*** were analyzed to answer the set of objectives and proposed research questions. The result indicates that from 2021 onwards, there was a significant wave of publications of papers about cheating and plagiarism.

*The results of the literature review suggest* that students have often broken the rules and regulations of the institutions they are enrolled in. Some students make it a habit to engage in dishonest behaviors like cheating and plagiarism ( Amzalag et al., 2021). Besides, cheating and plagiarism, students are also involved in other forms of academic dishonesty such as contract cheating ( Ali & Alhassan, 2021), collusion ( Chen, 2022), falsification ( Mâță et al., 2020), fabrication ( Khan, 2022), fraud, corruption, and unjustified absences. These are the top prevalent types of academic integrity violations in HEIs. Therefore, it has become challenging for higher educational institutions to maintain academic integrity and ensure quality education for all students.

There is a high contextual basis for the causes behind cheating and plagiarism and this differs, from one individual to another and from one nation to the next. Based on the study, there are several contributors to students' infractions of academic honesty and integrity and this includes peer pressure, internet availability, societal norms, and individual mindset ( Abbasi et al., 2021). These factors are categorized as individual factors, institutional factors, social, cultural, and technological factors which are summarized in the following paragraphs.

***Individual factors**:* individual factors may contribute to the student’s choice to breach academic honesty. It is related to students' motivation. About this, motivation is an English word that stems from the word, “moveo”, which means to arouse, urge someone to act, or annoy. In other words, the system can sometimes initiate, stimulate, and maintain a particular behavior among individuals ( Landa-Blanco et al., 2020; Usher, 2018). Motivation is a tool that can urge someone to act in a certain way and academic motivation is thus the root behind the behaviors relating to the performance and function of academic behavior ( Kuhlmann et al., 2023) like the low level of students’ efforts, management of workload among students, their selected activities and their persistence in their academic development.

A range of studies have explored the relationship between student motivation and academic dishonesty such as cheating and plagiarism. According to Landa-Blanco et al. (2020), academic dishonesty is negatively associated with intrinsic motivation and self-efficacy, and positively linked with motivation and extrinsic goal orientation. There is a significant connection between academic dishonesty and both intrinsic and extrinsic motivation. ( Abbasi et al., 2021) further delved into the role of personality traits in academic dishonesty, finding that impulsivity and fight-flight-freeze behaviors were positively associated with engagement in dishonest academic behaviors. These studies collectively suggest that both intrinsic and extrinsic motivation, as well as certain personality traits, play a role in influencing cheating and plagiarism.

***Institutional factors:*** This category of factors covers undermining practices to the academic integrity in the classroom, namely external assistance, collaboration, plagiarizing, and technology used for cheating. The environment in the classroom and the atmosphere within it reflects the students’ personalities and through the usage of academic materials, such an environment becomes a specific setting for learning advancement and promotion of creativity ( Thomas, 2020). Studies show that a positive classroom atmosphere supports the learning outcomes of the students in light of their success and content level ( Chang et al., 2018). Various physical environments, contexts, and cultures within which students learn are referred to as the academic environment (and such a learning environment, directly impacts the drive of the students to study.

***Social factors:*** A range of social factors have been identified as significant contributors to academic integrity violations such as competition, social rejection, and societal pressure while ( Șercan & Voicu, 2022) highlighted the influence of performance goals and social norms. These studies collectively underscore the complex interplay of social influences on academic integrity, suggesting the need for targeted interventions and support mechanisms. Moreover, cultural differences were also found to play a role, wit Roe (2022) and Nabee et al. (2020) both identifying the influence of perceived peer dishonesty and the acceptability of cheating, as well as the impact of cultural and psychological variables such as distress, perfectionism, and self-control. These findings underscore the complex interplay of social and individual factors in shaping academic dishonesty.

***Cultural factors:*** The origin of cultural factors is the home, and the main source is parental pressure. Generally, parents pressure their children towards academic excellence notwithstanding the latter’s abilities and capabilities. Hence, students may turn to academic integrity breaches to satisfy their parents ( Won & Lee, 2023). Studies showed that some students feel that obtaining high academic standards is a must to meet their parents’ expectations ( Farahat, 2022). Added to this, parents force their children to obtain high marks despite their actual ability to do so and as such, parental expectations could make students compromise their academic integrity. A related study by Rozar et al. (2020) revealed that plagiarizing attempts among students usually occur when faced with tough tasks when obtaining a good score would call for extra effort and time, and when there is pressure from family. Their belief in the impossibility of obtaining good scores in limited time drives students to engage in cheating after which this becomes a part of their culture. Bretag & Mahmud (2014). found that institutionalizing a culture in the academic policy and practice context can promote academic integrity among students. In other words, understanding the way academic culture influences the inclination of students to breach academic integrity is the key.

***Technological factors:*** Technology advancements have been dynamic over time and technology use among students has unfortunately taken a turn for the worst when it comes to cheating and stealing other’s work. In other words, ICTs have made it possible for students to easily plagiarize and cheat in HEIs. These infractions may also be brought on by improper and illegal ICT usage. It is thus important for students to understand and respect the rights and obligations of using and sharing intellectual property by adhering to the copyright and fair use, citing resources, obtaining or providing permission for use, steering clear of plagiarism, and understanding and using creative commons ( Coldwell-Neilson, 2020). Morrison (2018), stated that the California Model School Library Standards indicate the need for students to respect intellectual property its fair use, and public performance rights when it comes to media downloading and duplication.

Cheating and plagiarism are a recurrent issue in higher education institutions. The reasons for cheating and plagiarism vary from person to person. Higher education institutions are required to take preventive measures to stop cheating and plagiarism as they hurt institutions' reputations, undermine the institutional code of ethics, and sideline the sustainability of the ethics and morality of higher education institutions. There is no one-stop solution for reducing the reasons for cheating and plagiarism in higher education institutions. Therefore, higher education institutions need to adopt a adopt a comprehensive strategy to safeguard against student cheating and plagiarism behavior.

The initial step is to reform and reinforce academic honor code and ethical guidelines which every student must follow. Students need to be informed and aware of the appropriate and inappropriate conduct in the classroom and based on past studies, the majority of the learning institutions do not stress the importance of academic integrity, which provides the impression that it is not a crucial issue. Through academic policies, the academic integrity level of HEI students may be enhanced. Moreover, this honor code needs to be regularly reviewed and updated to adapt to the evolving landscape of academic dishonesty. At the same time, higher education institutions must promote a culture of ethical behavior and educate both students and faculty on the importance of maintaining integrity by implementing academic integrity awareness programs. New students can be the recipients of customized course to inculcate within them the basics when it comes to academic dishonesty and its consequences. Cooperation between teachers, writing center directors, library directors, and academic specialists to develop such a course for students could be the key (Darmansyah & Darman, 2022). New incoming students would be required to take the course to enhance their academic integrity in what could be a first-year requirement ( Stephens et al., 2021). HEIs need to play a key role in inculcating the habit of honesty, fair dealing, and integrity into students considering one of their major aims is to generate highly qualified graduates who can uphold the highest standards of integrity and professional ethics to contribute to society. Besides, higher education institutions must strengthen policy and invest resources to develop anti-plagiarism detection software using algorithms to scan and analyze submitted work, identify cases of plagiarism, and promote originality. Although the existing plagiarism prevention software (Turnitin, Authenticate, and Plagiarism Checker) has proven to be effective in the fight against academic cheating and plagiarism however this need to be modified further to detect AI generated contents. Furthermore, higher education institutions must introduce counselling and advisory services in shaping students' understanding of ethical boundaries. This counselling service will provide opportunities to understand the consequences of their actions for cheating and plagiarism. Fundamentally, the synergy of policy improvements, awareness initiatives, technical solutions and supportive advice will not only prevent cheating and plagiarism, but also improve academic integrity within higher education institutions ( Farahat, 2022). This creates a comprehensive framework that fosters genuine engagement in higher education institutions.

Implications of the study

Cheating and plagiarism are major issues in higher education institutions. The misuse of technology and artificial intelligence tools has made these problems even more challenging. Based on a comprehensive literature review, it is evident that higher education institutions must make it a priority to combat cheating and plagiarism. Additionally, it is essential to educate and raise awareness among students about the severe consequences that come with these actions.

## 6. Conclusions

Cheating and plagiarism are serious problems in higher education institutions around the world. This systematic literature review found that there are different forms of academic integrity violations in higher education institutions. Students cheating and plagiarism are the most common form of academic integrity violation.

Several factors contribute to this problem, including academic environment, cultural, social, and individual factors. The study also revealed that cheating and plagiarism are also caused by other variety of factors, including lack of student motivation to study, peer approval of cheating, laziness, strict submission deadline, course difficulty, lack of integrity awareness, and illegal use of cutting-edge technology, and increased pressure on students to improve their academic performance. The literature analysis also revealed that cheating and plagiarism increased further when higher education institutions moved to virtual assessments and evaluations during the Covid-19 pandemic. The introduction of electronic assessments has been found to increase the prevalence of cheating and plagiarism. Therefore, there is an urgent necessity to address this problem by designing effective policies and interventions.

Solving the issues related to cheating and plagiarism requires a comprehensive approach among faculty, administrators, and students is paramount. Moreover, universities need to create environments where students are motivated to learn and enhance ethical and moral values to become positive contributors to society. In such a learning environment, students are more likely to act and work with integrity and demonstrate the highest level of integrity. Besides, to improve students' academic integrity, educational institutions should invest in educational initiatives that promote ethical behavior, critical thinking, and the development of strong research and writing skills. Additionally, there is an urgent need to implement advanced plagiarism detection tools to make students understand their current level of skills and offer them a solution to develop skills and produce a revised version of the copy with an accepted version of plagiarism in academic writing. Overall, the results of this systematic literature review highlight the urgent need for higher education institutions to prioritize efforts to combat cheating and plagiarism and increase student knowledge and awareness about the consequences of cheating and plagiarism.

### Limitations of the study

This systematic literature review provided valuable insight into the prevalence of cheating and plagiarism in higher education institutions. However, there are certain limitations of this study. The main limitation is that relevant studies published in journals not indexed in Scopus are excluded. This can lead to biased literature selection and the loss of valuable articles from reliable journals that are not included in the Scopus database. Scopus primarily indexes English-language journals and may exclude valuable research published in other languages. This limitation can lead to an incomplete understanding of the global landscape of cheating and plagiarism in higher education, especially in regions where English is not the primary publication language.

Another limitation is the different research methods and definitions of cheating and plagiarism in studied studies. Different researchers have different approaches, which can make it difficult to accurately compare and generalize results. Differences in survey instruments, assessment methods, and cultural backgrounds can lead to inconsistencies in the reported prevalence, making it difficult to draw consistent conclusions. Despite these limitations, the Scopus framework provides a structured approach and allows access to a wide range of peer-reviewed literature. Future researchers should consider these limitations and take them into account when interpreting and generalizing the results of the review.

### Ethics and consent

Ethical approval and Consent were not required.

### Reporting guidelines

The Qualitative Data Repository: PRISMA checklist for ‘Cheating and plagiarism in higher education institutions (HEIs): A literature review’, ( Sozon et al., 2024) https://data.qdr.syr.edu/dataset.xhtml?persistentId=doi:10.5064/F6NEGI7I.

## Data Availability

No data are associated with this article. The Qualitative Data Repository: Extended data for ‘Cheating and plagiarism in higher education institutions (HEIs): A literature review’. https://www.doi.org/10.5064/F6NEGI7I ( Sozon et al., 2024). This project contains the following extended data:
1. A list of inclusion and exclusion criteria for the study. 2. Assessment of risk of bias for the included papers. 3. List of included papers for the study (n=45).
