# Supplementary material for: Cheating and plagiarism in higher education institutions (HEIs): A literature review
Source: F1000Res. 2024 Sep 26;13:788. Originally published 2024 Jul 10. [Version 2] doi: 10.12688/f1000research.147140.2 (PMC11489843; doi:10.12688/f1000research.147140.2)
Supplement: Peer review history

# Reviewer report: Reviewer response for version 2

**DOI:** 10.5256/f1000research.172032.r332655

**Published:** 2024-11-09

**Authors:** Amanda McKenzie

**Affiliations:** 1 University of Waterloo, Waterloo, Ontario, Canada

Literature reviews in niche fields such as academic integrity are invaluable and help identify strengths, weaknesses and trends in the current research and areas for future investigation.

 In this systematic review, I questioned why the terms cheating and plagiarism were the main focus (i.e., why weren’t other terms like academic misconduct more overtly included). I see that additional key terms (academic dishonesty, academic misconduct, academic integrity violation) were included in the database search but only cheating and plagiarism seem to be emphasized throughout the text. Just an observation.

 Also, it’s important not to conflate cheating and plagiarism as not all plagiarists are cheaters – unintentional plagiarism happens, and this does not equate to a lapse in morals. Moreover, cheating is an umbrella term that can result in overrepresentation in academic offence data. For example, plagiarism can be a subset of cheating. If an offence is labelled as both – the offence numbers will be higher than reality.

 The research questions outlined in this article are appropriate and the methods and inclusion/exclusion criteria are sound.

 The coding document only focused on bias domains. I would have liked access to their qualitative analysis files so I could see how they developed their themes and conclusions.  In the results section, I liked the way they took pre and post COVID impacts into consideration on the frequency of publication.

 The overall readability has some issues (see my minor comments below). A good proof-read  would be useful as there are spots where the sentences do not make sense, and words or pluralizations are missing. Examples, in the **Introduction**, in the second paragraph read: “ *students not considered as high-risk technology tool about cheat”. *I do not understand what this means. Also, “ *teachers find [it] hard to detect the authenticity of students assignments and exams*” – this sentence is missing the word “it”.

 There are also several instances of repetition or reiteration of the same points without introducing any new evidence. Specifically, the number of mentions of cheating and plagiarism throughout the text. The article would be more concise and have a stronger argument if some of these repeated statements and areas of duplicity were removed.

 In sum, I have reservations with the data and outcomes in this article. I have waivered between selecting “approved with reservations” and “not approved” given that I am the third person to do a review. However, it’s imperative that we have faith in the data and findings, and I am not confident with the way it has been presented, particularly the data in **Figure 9.** In addition, using references (i.e., Waigand, 2019) to support unsubstantiated findings is very concerning. Hence, my recommendation is “not approved” without mandatory revisions.

***The comments below are listed as they sequentially arise in the article. I have also denoted in each if they are major or minor comments.***

**Abstract**

 In the 1st paragraph, suggest rephrase to "Cheating and plagiarism **are **serious problems in HEIs" rather than **have** become serious problems. These offences have always been serious and are not new, however what is of concern is the rise in the number of incidents and ease at which these offences can be committed which has been exacerbated by technology, specifically the internet. **Minor**

 In the 3rd paragraph you refer to " *unethical application of AI tools*" - I found this too vague. Please define or spell out what you mean by AI as this acronym is widely used for both academic integrity **and** artificial intelligence - especially since either could possibly apply in this sentence. **Minor**

 Also in 3rd paragraph, last sentence in the abstract - I think you meant: "In a broader sense, all these factors can be classified into individual, social, cultural, institutional, and technological factors that contribute to these offences". Consider revising this sentence. **Minor**

 I would remove or revise reference to " *this*" problem in the 3rd and 4th paragraph because cheating and plagiarism are two separate things (or pluralize "this" to "these"). Alternately, together cheating and plagiarism are examples of academic misconduct and hence if referred to as academic misconduct then "this" problem is appropriate. **Minor**

 In the 4th paragraph, you write " *higher education institutions must develop anti-plagiarism detection software in order to detect plagiarism*" - do you perhaps mean HEIs should use anti-plagiarism detection software (rather than develop their own?). There are many products on the market that are readily available and have a solid reputation and business foundation rather than building your own detection tool. As an aside, such tools are also more accurately called text-matching or similarity tools. They only point to issues of copying and unoriginality but are not absolute evidence of plagiarism. This can only be confirmed by human review. Please review this section and clarify why you have included this suggestion with evidence. **Major**

 5th paragraph - suggest removing word " *solve*" and use "address". Cheating and plagiarism will never 100% be solved but we can take action to reduce it or dissuade such behaviour. **Minor**

**Introduction**

 1st paragraph - how did you come up with the figure of " *97%*" and who are the " *guardians in the United States*" (perhaps this is a term relevant in your context but unfamiliar to me?). I have concerns about this claim given that there is not clear process or evidence as to how you arrived at this percentage. Please clarify how this was determined and define what you mean by a “guardian”. **Major**

 2nd paragraph – suggest change " *recurrent occurrence*" to "proliferation" or "recurrence" and " *supports to violate*" to "can" violate academic integrity (it's all in how you use the tool). **Minor**

 3rd paragraph - you've mostly referred to HEIs however in this paragraph you specifically mention “ *universities*”. I suggest you remove that and use HEIs to be consistent and for broader applicability. **Minor**

 Last paragraph - " *This study will also explore the design and development of plagiarism detection software and innovation assessment systems that encourage critical thinking, originality, and ethical research practices*." This piece seems like a strange addition as developing software or assessment systems is not the focus of this paper nor was it an objective or research question. Moreover, there are several reputable products already on the market in this area (unless they are not available in the authors specific educational market) - it does not seem financially viable or logical to develop another product. I suggest that any reference to “ *explore the design and development of plagiarism detection software and innovation assessment systems*” be removed. **Major**

**Literature review**

 Paragraph 3 - last sentence has typos. “ *However, this problem bv hgbvbv cheating*.” **Minor**

 Paragraph 5 -

 “ *Plagiarism is the top frequently used method in academia which breaches the integrity of HEIs*”. Suggest rewrite to “Plagiarism is the most frequent breach of academic integrity in HEIs”. **Minor**

 Paragraph 5 – does “ *Information and communication technologies (ICT) development*” mean the same as artificial intelligence or generative AI? If not, how do they differ? Perhaps add an example to illustrate so it’s clear.

 Also, in paragraph 5 - the sentence " *a normal and decent person possessing morals and values would never engage in plagiarism behavior*" **is a falsehood **as research shows sometimes plagiarism is unintentional and is a writing problem rather than a err in morals. Any reference to being immoral if you plagiarize should be removed. **Major**

 6th paragraph - you mention " *colleges*" suggest replacing with HEIs to be consistent. **Minor**

 Also, where is the evidence to support this claim? " *Regardless of such technological detection of plagiarism, institutions in developed nations are still not leveraging effective plagiarism detection software*". How did you come to this conclusion – it’s not explicit. **Major**

 Paragraph 7 remove " *universities*" replace with HEIs. **Minor**

 I also suggest adding a reference (Guerrero-Dib et al., 2020) to Ozcan et al., 2022 regarding academic misconduct and later unethical issues in work. **Minor**

 Also in 7 ^th^ paragraph, the claim: “ *Furthermore, students' cheating and plagiarism may lead teachers, prospective students, and researchers to engage in similar inappropriate activities in their fields* ( Waigand, 2019).” Does not align with the reference provided. I reviewed this source and could not locate any indication or finding to support this statement. Hence, I believe this claim cannot be verified and should be revised with evidence or removed. **Major**

 Last paragraph - " *Furthermore, this study will reinforce the necessity to upgrade the existing plagiarism detection software and make it more diversified to detect AI and software-produced write-ups.* " This seems unwarranted and no evidence has been given to why this would be needed. The literature in this area suggests that AI detection tools are unreliable and that when students take GenAI created content and further manipulate it using other tools to make it sound more human or to contain a few errors – the GenAI detectors are rendered virtually useless. More reading in this area should be done before making the recommendation to include an AI detector. **Major**

**3.2.2 Exclusion criteria**

 While I am not experienced in a SLR, I would have liked a wider range of disciplines to be captured in the search rather than this criterion: *“EX6: The journal paper is not related to the Social Sciences, Arts and Humanities, and Psychology field.” *Given that the exploration of academic integrity occurs across many disciplines, and it is a relatively young and niche area of research – this criterion seems a bit limiting. We know that research has been published in other disciplines such as engineering and computer science. Although, I recognize that only 12 papers were eliminated for this exclusion. This comment is just for consideration – no revisions minor or major are required.

**Results**

**Figure 9.** The source of publications by nation seems to misrepresent the frequency of publications. If Indonesia has n=2 and Malaysia has n=1 - how can this area of the world have the darkest/highest score of publications at 4? (I only count 3). Also, Canada and the USA both have the same score (n=2) but they are different colours of blue (assuming my computer monitor is properly displaying the colours) - suggesting Canada has darker blue/more publications than the USA. Furthermore, Turkey (n=3), China (n=2) and Canada (n=2) are all the same colour ... which does not align with the data frequencies provided. Given the discrepancies above I selected only "partly" for both the "analysis/interpretations" and "conclusions" sections. These discrepancies are misrepresentations of the data and need to be resolved. **Major**

**Table 3** in the results – evidences that individual factors are the most prevalent which is what I would expect compared to the other factors listed.

 Just before **Table 4**, there is a typo: “ *Here are some common strategies that have been employe[d]*”. Please correct. **Minor**

**Discussion**

 Under **societal factors **there is a typo: “Moreover, cultural differences were also found to play a role, wit[h] Roe (2022) and Nabee et al. (2020)” **. **Please correct. **Minor**

 In **Technological Factors**, does ICTs mean the same as AI (artificial intelligence)? For consideration. In the same section, this should be rewritten: “ *internet and Internet and web-based unauthorized collaboration*.” Suggest removing the first instance of “internet”. **Minor**

**Limitations of existing tools in detecting cheating and plagiarism**: I would have expected to also see generative artificial intelligence listed here in addition to ghostwriters. For consideration.

 For consideration, as currently written, how does the subsection of **availability** differ from the section on **Internet and web-based unauthorized collaboration**? I would have expected some mention of the ease of access and proliferation of available tools in availability whereas, the unauthorized collaboration is the misuse of these tools.

 Cheating statistics are rarely measuring the same thing … (e.g., what Singh is measuring is completely different than the stats on ghostwriting that Curtis mentions). I would recommend adding more current sources on the prevalence of cheating behaviours rather than just using Singh et al. (2016) as this is an old source and there are many other recent sources that could be noted. Also, be aware that these “cheating” statistics might include plagiarism. You might want to consider adding separate plagiarism figures here too.  

 Regarding this statement: *“Although the existing plagiarism prevention software (Turnitin, Authenticate, and Plagiarism Checker) has proven to be effective in the fight against academic cheating and plagiarism however this need to be modified further to detect AI generated contents.” *I have already mentioned that AI detectors exist in some of these products and there is research that discusses the utility of such tools. I suggest reviewing this literature and reconsidering this suggestion.

**Conclusions**

 For consideration, when you state that cheating (75.55%) and plagiarism (42.22%) as the most reported violations can you be sure that the cheating reporting in the articles is distinctly separate from plagiarism (i.e., that plagiarism wasn’t included in the cheating figures)?

 I do not understand what you mean with this: “ *Additionally, there is an urgent need to implement advanced plagiarism detection tools to make students understand their current level of skills and offer them a solution to develop skills and produce a revised version of the copy with an accepted version of plagiarism in academic writing*.”  Do you mean GenAI detection when you state “ *advanced plagiarism detection tools”?* Also, the number (a percentage) generated by the AI detection tool in Turnitin is not visible to the student – so I’m not sure how that would improve their skills. This would require instructors to somehow share the number with the students and engage in conversation about the number. In addition, what do you mean by *“accepted version of plagiarism in academic writing*.”  This does not make sense to me. Please review and revise. **Minor.**

 Upon reading the entire paper, **I suggest removing reference to**:

 “ *Furthermore, higher education institutions must develop anti-plagiarism detection software in order to detect plagiarism and aid students in improving academic writing and paraphrasing approaches*.” which appears in the **Abstract. **This suggests HEIs must build their own product which is not true. **Major**

 Also, “ *This study will also explore the design and development of plagiarism detection software and innovation assessment systems that encourage critical thinking, originality, and ethical research practices*.“ which appears in the **Introduction**. Neither design nor development of either product is discussed in this article. **Major**

Are the rationale for, and objectives of, the Systematic Review clearly stated?

Yes

Is the statistical analysis and its interpretation appropriate?

Partly

If this is a Living Systematic Review, is the ‘living’ method appropriate and is the search schedule clearly defined and justified? (‘Living Systematic Review’ or a variation of this term should be included in the title.)

Not applicable

Are sufficient details of the methods and analysis provided to allow replication by others?

Partly

Are the conclusions drawn adequately supported by the results presented in the review?

Partly

Reviewer Expertise:

Academic integrity, quality assurance, generative artificial intelligence and academic integrity.

I confirm that I have read this submission and believe that I have an appropriate level of expertise to state that I do not consider it to be of an acceptable scientific standard, for reasons outlined above.

## References

[1] : Impact of academic integrity on workplace ethical behaviour. *International Journal for Educational Integrity* .2020;16(1) : 10.1007/s40979-020-0051-3 10.1007/s40979-020-0051-3

# Reviewer report: Reviewer response for version 2

**DOI:** 10.5256/f1000research.172032.r332653

**Published:** 2024-11-04

**Authors:** Som Nath Ghimire

**Affiliations:** 1 Faculty of Social Sciences and Education,, Nepal Open University, Lalitpur, Nepal

**Dear authors,**

 Congratulations on your contribution to the field under your scrutiny, which requires continued attention. I believe that your paper could be valuable for readers and future researchers to conveniently grasp the existing debates on cheating and plagiarism in higher education as well as guide them for any future endeavors. However, I have a few suggestions detailed below, hoping that addressing these concerns would significantly benefit and enhance the specificity, comprehensibility, and rigor of your study.    

**Title**

 Consider removing "HEIs" from the title. Introduce this abbreviation within the text after defining the term in full.

**Overall comments for Introduction, Literature Review, Results, and Discussion**

 Please clearly differentiate between cheating and plagiarism. Similarly, distinctions among their subcategories need to be fully articulated in the literature review. This would benefit from clarification on these distinctions even for Table 2 of your result section. Secondly, the study does not explore potential differences in the factors contributing to each type of academic dishonesty or the specific prevention strategies tailored for each. Reviewing the literature for these variations could add depth, especially in addressing any differences in factors and strategies for mitigating cheating and plagiarism in higher education. A more sophisticated analysis could further enhance the rigor of the study. Please consider a substantive revision of the introduction and literature review to strengthen argumentative depth.

 Doing this would certainly justify the purpose of your research question 2 **—"What are the top prevalent types of plagiarism and cheating in HEIs?”**

 Indicating the vagueness and difficulties regarding this issue only in the limitation section would not fully justify the author’s inability to draw the required distinctions for the purpose and scope of the study.

**Table 2**

 The categories in Table 2 would benefit from additional context to clarify their distinctions. For example, defining the differences between “cheating” and “contract cheating,” or “copying” and “collusion,”, along with others, would provide a clearer framework for readers. It appears the table lists terms used by the reviewed studies without classifying them into distinct subcategories. Defining cheating and plagiarism as separate forms of academic dishonesty and further dividing each into subcategories could make the table more coherent and informative.

**Table 3**

 Consider providing clarity on specific individual, institutional, social, cultural, and technological factors identified in the reviewed literature. Expanding the mind map to include related subcategories would enhance readers’ understanding and offer a more comprehensive view of the factors influencing cheating and plagiarism.

**Table 4**

 Please clarify how academic integrity awareness programs, counseling, and guidelines are distinct from one another. For example, isn’t is possible that the concept of guidelines overlaps with academic integrity policies or honor codes? Clearly defining each strategy and its subcategories would help readers understand the unique contributions of each approach to preventing cheating and plagiarism in higher education.

 Revising your Table 3 and Table 4 would certainly strengthen the relevance of your research questions 3 and 4: “ **What factors contribute to the prevalence of cheating and plagiarism in Higher education institutions? What strategies have been employed to reduce the prevalence of cheating and plagiarism in higher education?”**

**Discussion**

 In its current form, there is a huge gap between your results and the discussion section since your findings do not clearly articulate the ideas discussed. In the discussion section, start with a concise summary of the results and then explore each finding in greater depth, particularly concerning factors and prevention strategies for both cheating and plagiarism. To strengthen this analysis, consider incorporating recent studies on factors influencing cheating and plagiarism and effective prevention strategies, as well as any limitations and challenges these studies have identified. This approach could offer a more comprehensive view and enhance the relevance of your findings.

 I look forward to reading the revised version of this paper. Best wishes.

Are the rationale for, and objectives of, the Systematic Review clearly stated?

Partly

Is the statistical analysis and its interpretation appropriate?

Partly

If this is a Living Systematic Review, is the ‘living’ method appropriate and is the search schedule clearly defined and justified? (‘Living Systematic Review’ or a variation of this term should be included in the title.)

Not applicable

Are sufficient details of the methods and analysis provided to allow replication by others?

Partly

Are the conclusions drawn adequately supported by the results presented in the review?

Partly

Reviewer Expertise:

Higher education, academic integrity, technology in education, critical discourse studies, gender studies, media and surveillance, and rhetoric and communications.

I confirm that I have read this submission and believe that I have an appropriate level of expertise to confirm that it is of an acceptable scientific standard, however I have significant reservations, as outlined above.

# Reviewer report: Reviewer response for version 2

**DOI:** 10.5256/f1000research.172032.r327179

**Published:** 2024-10-18

**Authors:** Ceceilia Parnther

**Affiliations:** 1 Le Moyne College, Syracuse,, NY, USA

Thank you for your thoughtful attention to these revisions.

Are the rationale for, and objectives of, the Systematic Review clearly stated?

Yes

Is the statistical analysis and its interpretation appropriate?

Not applicable

If this is a Living Systematic Review, is the ‘living’ method appropriate and is the search schedule clearly defined and justified? (‘Living Systematic Review’ or a variation of this term should be included in the title.)

Not applicable

Are sufficient details of the methods and analysis provided to allow replication by others?

Partly

Are the conclusions drawn adequately supported by the results presented in the review?

Partly

Reviewer Expertise:

Academic integrity, student success, leadership

I confirm that I have read this submission and believe that I have an appropriate level of expertise to confirm that it is of an acceptable scientific standard.

# Reviewer report: Reviewer response for version 1

**DOI:** 10.5256/f1000research.161295.r308208

**Published:** 2024-09-09

**Authors:** Ceceilia Parnther

**Affiliations:** 1 Le Moyne College, Syracuse,, NY, USA

Thank you for your submission. Your global study is helpful and needed. The rationale and objectives are clear , the analysis section is very thorough and complete, and the four research questions are straightforward. Your methodological choices and descriptions are clear and maintain rigor. Your conclusions are logical and suggestions, while broad, align with the findings.

 Several uncited studies popularized the PRISM technique in academic integrity research before 2021 . I suggest mentioning one or more given the shift in approach.

 In terms of added clarity, defining subsections in the technological factors section would be helpful. In addition the cheating types discussion section would benefit from with additional context. It is surprising that only one study uses the term academic misconduct. Providing more context on the factor types, specifically institutional and social factors as described would provide a more comprehensive understanding of the topic. I suggest you consider the absence of findings a finding, and explaining what is will yield stronger future research and implications. Please consider connecting the findings to the research questions. I also recommend potential elaboration of prior literature specific to cheating. Thank you!

Are the rationale for, and objectives of, the Systematic Review clearly stated?

Yes

Is the statistical analysis and its interpretation appropriate?

Not applicable

If this is a Living Systematic Review, is the ‘living’ method appropriate and is the search schedule clearly defined and justified? (‘Living Systematic Review’ or a variation of this term should be included in the title.)

Not applicable

Are sufficient details of the methods and analysis provided to allow replication by others?

Partly

Are the conclusions drawn adequately supported by the results presented in the review?

Partly

Reviewer Expertise:

Academic integrity, student success, leadership

I confirm that I have read this submission and believe that I have an appropriate level of expertise to confirm that it is of an acceptable scientific standard, however I have significant reservations, as outlined above.

# Response

**Published:** 2024-09-17

**Authors:** Md Sozon

**Affiliations:** FAM, Universiti Tunku Abdul Rahman, Petaling Jaya, Selangor, Malaysia

**Dear esteemed reviewer**,

 Thank you so much for reviewing our paper. We appreciate your time and effort in providing constructive feedback and suggestions. Your input has greatly improved the paper. We are pleased to inform you that we have incorporated your suggestions into version II of the paper. Kindly, let us know if any further revisions are required.

 Thank you once again for your valuable input.

 Sincerely,

 Md. Sozon [PhD Candidate] Faculty of Accountancy and Management, Universiti Tunku Abdul Rahman, Malaysia

**Reviewer Comment 1: Several uncited studies popularized the PRISM technique in academic integrity research before 2021. I suggest mentioning one or more given the shift in approach.**

**Our reply: We have included one pertinent research paper in the study, as per your suggestion.**

**Revised text: **After conducting a comprehensive review of the existing literature, it is clear that the PRISM technique has become widely popular in the field of academic integrity. Zawacki-Richter's (2020) study, in particular, has been instrumental in bringing clarity, replicability, and a comprehensive synthesis of the literature to academic integrity research.

1. Zawacki-Richter, O., Kerres, M., Bedenlier, S., Bond, M., & Buntins, K. (2020). Systematic reviews in educational research: Methodology, perspectives and application (p. 161). Springer Nature.
https://library.oapen.org/handle/20.500.12657/23142

**Reviewer Comment 2:  In terms of added clarity, defining subsections in the technological factors section would be helpful. In addition the cheating types discussion section would benefit from with additional context.**

**Our reply: We have revised this section to enhance its professional tone. Firstly, we offer a comprehensive definition of the technology and its significant impact on the prevalence of cheating and plagiarism in higher education. Following that, we delve into the critical technological factors in four distinct subsections. Lastly, we support each subsection by incorporating relevant statistics and citing credible sources.**

 Technology advancements have been dynamic over time and technology use among students has unfortunately taken a turn for the worst when it comes to cheating and stealing other’s work. In other words, ICTs have made it possible for students to plagiarize and cheat in HEIs easily. These infractions may also be brought on by improper and illegal ICT usage. It is thus important for students to understand and respect the rights and obligations of using and sharing intellectual property by adhering to the copyright and fair use, citing resources, obtaining or providing permission for use, steering clear of plagiarism, and understanding and using creative commons ( Coldwell-Neilson, 2020). Morrison (2018), stated that the California Model School Library Standards indicate the need for students to respect intellectual property its fair use, and public performance rights when it comes to media downloading and duplication.

 Cheating and plagiarism through the misuse of technology can be further divided into three subsections such as the availability of computer and& AI tools, limitations of existing tools in detecting cheating and plagiarism, internet and Internet and web-based unauthorized collaboration

**Availability of computer and& AI tools**: Computer and artificial intelligence tools have been shown to have a double-edged effect on higher education institutions. Recent research conducted by Hasanein & Sobaih (2023) has highlighted that students frequently misuse these tools, particularly ChatGPT, within the realm of higher education. Consequently, there has been a notable increase in instances of AI-driven cheating, plagiarism, contract cheating, and various forms of academic dishonesty, including falsification, collusion, and unauthorized collaboration. These challenges present significant obstacles to maintaining academic integrity (Wang & Cornely, 2023).

**Limitations of existing tools in detecting cheating and plagiarism**: The study conducted by Do Ba (2017) reported that higher education institutions are applying software and technological tools, like Turnitin, to assess the similarity index of students' written assignments/reports. However, the study conducted by Lines (2016) highlighted that these tools are often incapable of detecting assignments and reports written by ghostwriters. Therefore, the students who engage in committing cheating and plagiarism often go undetected because of the limitations of the existing tools for detecting such behavior.

**Internet and web-based unauthorized collaboration: **The study conducted by Wang & Cornely (2023) reported that students frequently engage in unauthorized Internet and web-based collaboration while completing their reports, theses, and assignments. In this connection, Tabsh (2019) emphasized that this type of unauthorized collaboration encourages cheating and plagiarism and diminishes the overall learning outcomes in higher education. Moreover, the unapproved and dishonest behaviors exhibited by students have raised concerns about data security and privacy. Therefore, it is necessary for higher education institutions to promptly take action to reduce these unethical student behaviors.

**Newly added references:**

 Hasanein, A. M., & Sobaih, A. E. E. (2023). Drivers and consequences of ChatGPT use in higher education: Key stakeholder perspectives. *European journal of investigation in health, psychology and education*, *13*(11), 2599-2614. . https://doi.org/ 10.3390/ejihpe13110181

 Wang, J., & Cornely, P. R. (2023, November). Addressing Academic Misconduct in the Age of ChatGPT: Strategies and Solutions. In *Proceedings of the 2023 7th International Conference on Education and E-Learning* (pp. 19-25). https://doi.org/10.1145/3637989.3638014

 Do Ba, K., Do Ba, K., Lam, Q. D., Le, D. T. B. A., Nguyen, P. L., Nguyen, P. Q., & Pham, Q. L. (2017). Student plagiarism in higher education in Vietnam: an empirical study. *Higher Education Research & Development*, *36*(5), 934-946. https://doi.org/10.1080/07294360.2016.1263829

 Lines, L. (2016). Ghostwriters guaranteeing grades? The quality of online ghostwriting services available to tertiary students in Australia. *Teaching in Higher Education*, *21*(8), 889-914. https://doi.org/10.1080/13562517.2016.1198759

 Tabsh, S. W., El Kadi, H. A., & Abdelfatah, A. S. (2019, April). Faculty perception of engineering student cheating and effective measures to curb it. In *2019 IEEE Global Engineering Education Conference (EDUCON)* (pp. 806-810). IEEE.

 Reviewer Comment 3: **It is surprising that only one study uses the term academic misconduct. Providing more context on the factor types, specifically institutional and social factors as described would provide a more comprehensive understanding of the topic.**

 Cheating and plagiarism continue to be persistent issues in higher education. A study by Singh et al (2016) revealed a prevalent occurrence of cheating among undergraduates. Approximately 39% of participants admitted to cheating on tests, and a majority of 62% admitted to cheating on written assignments. Among graduate students, 17% admitted to cheating on tests, while 40% admitted to cheating on written assignments. Furthermore, according to Curtis et al. (2021), the percentage of Australian students who buy assignments from ghostwriting services is estimated to be 7.9%, which is considerably higher than previous self-reported numbers.

 Previous research conducted by Cuadrado et al. (2019) has shown that these acts not only hinder students' learning outcomes but also damage the reputation of educational institutions. Moreover, students' cheating and plagiarism incidents undermine the institutional code of ethics and jeopardize the sustainability of ethical values and principles within these institutions.

 The causes of cheating and plagiarism can vary from person to person. According to Sozon et al. (2024), individual causes include laziness, lack of motivation for learning, and insufficient time to study. In terms of institutional factors, Solmon (2018) revealed that a poor academic integrity policy, ineffective implementation of an honor code, and a lack of technological tools for detecting cheating and plagiarism contribute to the issue.  Tanveer et al. (2012). Additionally, Sozon et al. (2024) highlighted those social factors, such as peer influences, pressure to achieve higher grades, intense competition, cultural norms, and the perceived acceptance of cheating, also play significant roles in shaping student behavior.

 There is no one-stop solution for reducing the reasons for cheating and plagiarism in higher education institutions. Therefore, higher education institutions need to adopt a comprehensive strategy to safeguard against student cheating and plagiarism behavior. Parnther (2022) recommends increasing student engagement, providing more opportunities for part-time faculty to share and disseminate ideas, demonstrating student learning, and establishing a clear policy and shared mission to address the issue of academic misconduct, such as cheating and plagiarism, in higher education institutions.

 The scope of this study covers various forms of academic integrity violations, from classic exam cheating to sophisticated cases of plagiarism made possible by digital technology. It is crucial to recognize that combating academic misconduct such as cheating, and plagiarism requires a variety of approaches. While investigating academic misconduct in higher education, Parnther (2020) conducted a study to identify the characteristics of students who engage in academically dishonest behaviors and suggested best practices and challenges in preventing academic misconduct and promoting academic integrity.

**Newly added references:**

 Singh, P., Thambusamy, R. X., & Druckman, Z. A. (2016). Insidious, invasive, invisible: academic dishonesty and on-going assessments in higher education. *The European Journal of Social & Behavioural Sciences*. DOI: 10.15405/ejsbs.193

 Curtis, G. J., McNeill, M., Slade, C., Tremayne, K., Harper, R., Rundle, K., & Greenaway, R. (2022). Moving beyond self-reports to estimate the prevalence of commercial contract cheating: An Australian study. *Studies in Higher Education*, *47*(9), 1844-1856. DOI: 10.1080/03075079.2021.1972093

 Cuadrado, D., Salgado, J. F., & Moscoso, S. (2019). Prevalence and correlates of academic dishonesty: Towards a sustainable university. *Sustainability*, *11*(21), 6062. https://doi.org/10.3390/su11216062

 Sozon, M., Chuan, S. B., Fong, P. W., Alkharabsheh, O. H. M., & Rahman, M. A. (2024). Factors influencing academic dishonesty among undergraduate students in higher education institutions (HEIs) in Malaysia. *Journal of Management Info*, *11*(2), 137-159. DOI: https://doi.org/10.31580/jmi.v11i2.3039

 Solmon, M. A. (2018). Promoting academic integrity in the context of 21st century technology. *Kinesiology Review*, *7*(4), 314-320. DOI: https://doi.org/10.1123/kr.2018-0042

 Tanveer, M. A., Gill, H., & Ahmed, I. (2012). Why business students cheat? A study from Pakistan. *American Journal of Scientific Research*, *78*, 24-32.

Sozon, M., Sia, B.C., Pok, W.F. and Alkharabsheh, O.H.M. (2024), "Academic integrity violations in higher education: a systematic literature review from 2013–2023", *Journal of Applied Research in Higher Education* , Vol. ahead-of-print No. ahead-of-print. https://doi.org/10.1108/JARHE-12-2023-0559

 Parnther C. (2020). Academic Misconduct in Higher Education: A Comprehensive Review. *johepal*. *1*(1), 25-45. doi: 10.29252/johepal.1.1.25 URL: http://johepal.com/article-1-31-en.html

 Parnther, C. (2022). The Rise of Contract Cheating in Graduate Education. In: Eaton, S.E., Curtis, G.J., Stoesz, B.M., Clare, J., Rundle, K., Seeland, J. (eds) Contract Cheating in Higher Education. Palgrave Macmillan, Cham. https://doi.org/10.1007/978-3-031-12680-2_17

**Reviewer Comment 4: I suggest you consider the absence of findings a finding, and explaining what is will yield stronger future research and implications.**

 Our Reply: The absence of significant findings can provide valuable insights by revealing gaps in the literature. These gaps can then be used to guide future research and improve academic integrity.

**Reviewer comment 5:Please consider connecting the findings to the research questions. I also recommend potential elaboration.**

 Our reply: We have expanded on the findings pertaining to the research questions and additionally provided potential explanations.

  Thank you

# Reviewer report: Reviewer response for version 1

**DOI:** 10.5256/f1000research.161295.r311995

**Published:** 2024-08-20

**Authors:** Adva Margaliot

**Affiliations:** 1 Achva Academic College,, Arugot,, Israel

The paper clearly stated the importance of the need for this wide literature review of Plagiarism and Cheating in Higher education institution.

 The researchers did very wide literature scanning and convinced by the centric issue and its future influencing on the skills and the performances of the generation of students that uses the affordance of technology smart tools, they provide the high scale of the phenomena of cheating and Plagiarism at HEIs across the globe.

 The spread of such phenomena is well expressed and well examined along with this paper.

 The discussion section well detailed the phenomena as well as the urgent need to find some new directions so as to connect between the theoretical versus practical knowledge construction, so that academic knowledge construct will be better realize as relevant and worth for upload the self effectiveness for the promotion of livelihood.

Are the rationale for, and objectives of, the Systematic Review clearly stated?

Yes

Is the statistical analysis and its interpretation appropriate?

Yes

If this is a Living Systematic Review, is the ‘living’ method appropriate and is the search schedule clearly defined and justified? (‘Living Systematic Review’ or a variation of this term should be included in the title.)

Yes

Are sufficient details of the methods and analysis provided to allow replication by others?

Yes

Are the conclusions drawn adequately supported by the results presented in the review?

Yes

Reviewer Expertise:

Integrity & creativity; Creativity in teachers' education; Online learning and teaching

I confirm that I have read this submission and believe that I have an appropriate level of expertise to confirm that it is of an acceptable scientific standard.
